# Antibacterial Activity of Dihydroquercetin Separated from Fructus *Polygoni orientalis* against *Clavibacter michiganensis* subsp. *sepedonicus* via Damaging Cell Membrane

**DOI:** 10.3390/foods13010023

**Published:** 2023-12-20

**Authors:** Jin Cai, Shiqin Wang, Qi Wang

**Affiliations:** 1 Institute of Applied Chemistry, Shanxi University, Taiyuan 030006, China; 2 Morden Research Center for Traditional Chinese Medicine, Shanxi University, Taiyuan 030006, China; wangshiqin12247@163.com; 3 The Key Laboratory of Chemical Biology and Molecular Engineering of Ministry of Education, Shanxi University, Taiyuan 030006, China; 4 School of Life Science, Shanxi University, Taiyuan 030006, China; wangqi@sxu.edu.cn

**Keywords:** *Clavibacter michiganensis* subsp. *sepedonicus*, Fructus *Polygoni orientalis*, antibacterial activity, isolation, identification, cell membrane damage

## Abstract

The yield and quality of potato can be severely affected by bacterial ring rot, which is caused by *Clavibacter michiganensis* subsp. *sepedonicus* (Cms). Recently, using natural compounds to control bacteria has received more attention. In this study, five antibacterial compounds from ethyl acetate (EtOAc) extract of Fructus *Polygoni orientalis* (FPO) against Cms were isolated and the most active compound was screened. Five active compounds were identified as 3,3′-di-*O*-methylellagic acid (**1**), 3,3′-di-*O*-methylellagic acid-4-*O*-*β*-*D*-glucopyranoside (**2**), dihydroquercetin (**3**), protocatechuic acid (**4**) and quercetin (**5**). Compound **3** (dihydroquercetin, DHQ) was confirmed as the most active compound. The diameter of inhibition zone (DIZ), minimum inhibitory concentration (MIC), protective efficiency and curative efficiency of DHQ were 22.50 mm, 0.313 mg/mL, 84.49% and 79.63%, respectively, which exceeded these of thiophanate-methyl (TM) in antibacterial activity assays; this indicated that DHQ had satisfactory antibacterial activities against Cms in vitro and in vivo. Results of cell membrane damage assessments indicated that DHQ could reduce membrane potential (MP), disrupt the cell membrane integrity, and promote the leakage of nucleic acids and proteins. Overall, these findings suggested that DHQ could serve as a promising lead molecular against Cms, which could provide a basis for its further derivatization.

## 1. Introduction

Potato is native to South America and widely cultivated in about 160 countries and regions [1,2]. In 2021, China had the largest potato planting area (5.78 million hectares), accounting for 25.1% of the global potato production [3]. As a nutritious food, it is enriched with carbohydrates and dietary fiber [4]. It also can provide the human body with various minerals and vitamins [4]. In addition, potato has the reputation of “the king of vegetables” [5]. However, the yield and quality of potato can be seriously affected by bacterial ring rot [6]. This devastating disease is caused by *Clavibacter michiganensis* subsp. *sepedonicus* (Cms), which is one of the most important pathogens of potatoes [7,8]. Cms multiplies very quickly and can survive for many years on contaminated surfaces [9,10]. The main symptoms of this disease usually include leaves wilt, vascular tissue discoloration and milk droplets of bacterial slime [11]. Each year, Cms has caused losses of up to 60% in potatoes [12].

So far, the control of bacterial ring rot of potato usually relies on the application of chemical bactericides, such as thiophanate-methyl. However, the heavy use of chemical bactericides not only causes environmental pollution but also adversely impacts on human health [13]. Therefore, there is an urgent need to search for eco-friendly and safe antibacterial substances to control Cms, which would help to improve the yield and quality of potatoes. Due to the biodegradability and safe nature of plant-based compounds [14], they have attracted more attention. Currently, many researchers have reported the isolation of plant-derived compounds with antibacterial activity, such as quercetin-3-rutinoside from *Ficus sycomorus* and stigmasterol from *Monsonia angustifolia* [15,16]. Hence, plant-based compounds are a promising source for discovering and developing alternative antibacterial agents.

*Polygonum orientale* L., also called Hongcao, is classified as Polygonaceae. Its fruits are referred to as Fructus *Polygoni orientalis* (FPO) [17]. *P. orientale* originated from Australia and China, and is widely distributed in China [18]. As a traditional Chinese medicinal herb, the whole plant of *P. orientale* can be used as medicine to treat various diseases, including muscle injuries and coronary heart disease [19]. PFO has been listed in Chinese pharmacopoeia with the treatment of cardiomyopathy, swelling and hypertension [20]. *P. orientale* also has been used as food [18]. People have cultivated it as a stir-fry and soup ingredient because of its fragrant and spicy taste [21]. In addition, some studies have reported that *P. orientale* extracts show antibacterial activity against *Bacillus megateriumand*, *Escherichia coli* and *Botrytis cinerea* [22,23]. Therefore, it is very promising to isolate antibacterial compounds from FPO against Cms.

This was the first time that compounds that are antibacterial and active against Cms have been isolated from FPO. Firstly, antibacterial chemical compounds against Cms were isolated from FPO and identified, and the most active compound was screened using an agar well-diffusion method. Next, the antibacterial properties of the most active compound were compared with positive drug, TM, through measuring DIZ, MIC and control efficiency in both in vitro and in vivo experiments. Finally, the damage by which the most active compound acts on the membrane was evaluated by measuring membrane potential, cell membrane integrity and leakage of nucleic acids and proteins. This study was performed in order to determine the most active compound as a potential lead in molecular studies and to provide a basis for developing a series of new, safe and efficient compounds that act against Cms.

## 2. Materials and Methods

### 2.1. Plant Materials

From September to October 2018, the ripe fruits of *P. orientale* (FPO) were collected from aboveground regions in Taiyuan, China. The collected FPO was washed repeatedly to remove impurities. After drying at 30 °C, the samples were crushed to obtain 20 kg of powder.

### 2.2. Bacterial Strain

Cms (code: C. m. s.) was provided by Tingchang Zhao, a researcher at the Chinese Academy of Agricultural Sciences. Cms is an aerobic bacterium with spherical or rod-shaped cells [24]. Its storage and incubation temperatures were 4 °C and 28 °C, respectively. The Cms medium was composed of distilled water, peptone (10 g/L), sodium chloride (5 g/L), agar (15~20 g/L), and beef extract (5 g/L), pH = 7. All bacteria were deactivated through sterilization after the experiment.

### 2.3. Reagents

Ethanol, petroleum ether, *n*-butanol (BuOH), ethyl acetate (EtOAc), dimethyl sulfoxide (DMSO) and dichloromethane (CH_2_Cl_2_) were of analytical grade (Tianjin Tianli Chemical Reagent Co., Ltd., Tianjin, China). Analytical-grade chloroform (CHCl_3_) and methanol (MeOH) were, respectively, obtained from Beijing Chemical Works, Beijing, China and Tianjin Guangfu Technology Development Co., Ltd., Tianjin, China. DMSO-*d*6 and methanol-*d*4 were purchased from Qingdao Tenglong Weibo Technology Co., Ltd., Shandong, China. TM was obtained from Pilarquim (Shanghai) Co., Ltd., Shanghai, China. Sodium hypochlorite (NaClO) was obtained from Tianjin Damao Chemical Rwagent Factory, Tianjin, China. Phosphate-buffered saline (PBS) was obtained from Beijing Solarbio Science & Technology Co., Ltd., Beijing, China. Rhodamine 123 was purchased from MedChemExpress. Propidium iodine (PI) staining kit was provided by Sangon Biotech (Shanghai) Co., Ltd., Shanghai, China.

### 2.4. Preparation of Plant Extracts

The air-dried and powdered FPO (20 kg) was extracted through reflux in ethanol at 80 °C with a solid/liquid ratio of 1:10 (g/mL) for 6 h. After filtering, the ethanol phase was evaporated to obtain a crude extract. Through the sequential extraction method [16], the crude extract was sequentially partitioned with four different polar solvents to obtain M1 (petroleum ether phase, 26 g), M2 (CHCl_3_ phase, 61 g), M3 (EtOAc phase, 222 g), M4 (BuOH phase, 359 g) and M5 (water phase, 296 g).

### 2.5. Antibacterial Potential Analysis

The antibacterial potential of each partition extract was tested using an agar well-diffusion method [25] to screen the highest activity partition extract. Each partition extract was prepared in a 15 mg/mL solution by dissolving in 60% DMSO. The control was 60% DMSO. The Cms strains were cultured in medium at 28 °C for 12 h and 120 rmp. Afterwards, Cms solution was diluted with medium to gain a Cms suspension of 10^6^ CFU/mL for preservation. Then, 200 μL of Cms suspension was uniformly spread onto plates. The wells (10 mm) were punched out of the plates and the wells were filled with 200 μL of the above extract (15 mg/mL) and 60% DMSO. The plates were incubated at 28 °C for 24 h. Finally, the values of DIZ were measured.

### 2.6. Isolation and Identification of Active Compounds from EtOAc Phase

According to the highest antibacterial activity against Cms, the EtOAc phase (M3) was chosen to isolate through a bioassay-guided method; the most active compound was screened for action against Cms. The isolation scheme of the active compounds from M3 is presented in Figure S1. M3 (202 g) was isolated using a polyamide column, eluting with CH_2_Cl_2_–MeOH gradient (100:0–0:100) to gain nine fractions (Ⅰ–Ⅸ). Based on a bioassay-guided method, fractions Ⅰ, Ⅲ, Ⅵ and Ⅶ exhibited remarkable inhibitory effects against Cms. Then, these four fractions were further isolated to identify the active antibacterial compounds. Fraction Ⅰ (1.92 g) was separated by a polyamide column, eluting with CH_2_Cl_2_–MeOH gradient (100:0–0:100) to gain eleven subfractions (Ⅰ-1–Ⅰ-11). Subfraction Ⅰ-8 was purified on a polyamide column using CH_2_Cl_2_–MeOH (100:6.5) to yield active compound **1**. Fraction Ⅲ (2.4 g) was fractioned using an octadecyl silica (ODS) column with MeOH–H_2_O (20:80–40:60) to afford active compound **2**. Fraction Ⅵ (9 g) was separated on an ODS column using MeOH–H_2_O (30:70–100:0) as eluent to obtain seven subfractions (Ⅵ-1–Ⅵ-7). Subfraction Ⅵ-2 was purified through recrystallization from MeOH–H_2_O to yield active compound **3**. Fraction Ⅶ (2.73 g) was separated on an ODS column through eluting with MeOH–H_2_O (20:80–100:0) to obtain five subfractions (Ⅶ-1–Ⅶ-5). The further crystallization of subfraction Ⅶ-1 and Ⅶ-4 from MeOH–H_2_O yielded active compounds **4** and **5**, respectively. Their nuclear magnetic resonance (NMR) spectra and electrospray ionization mass spectra (ESI-MS) were performed on a Bruker Avance III HD 600MHz spectrometer (Bruker, Switzerland) and Thermo Scientific Q Exactive mass spectrometer (ThermoFisher Scientific, Waltham, MA, USA), respectively. The DIZs of compounds **1–5** were tested using the agar well-diffusion method.

### 2.7. In Vitro and In Vivo Antibacterial Activity Evaluation

#### 2.7.1. Determination of DIZ

DHQ was dissolved in 60% DMSO to obtain a 15 mg/mL solution. The positive control was treated with 15 mg/mL TM and the negative control was treated with 60% DMSO. The determination of DIZ was same as the method in Section 2.5. The concentration of the Cms suspension was 10^8^ CFU/mL.

#### 2.7.2. Determination of MIC

Microdilution method was adopted to measure the MIC of DHQ [26]. The DHQ solution (10 mg/mL) was prepared by culture medium containing 3% DMSO. Two-fold serial dilutions of DHQ solution were added into wells of 96-well plate at the final concentrations of 10~0.01 mg/mL. The 3% DMSO was used as the negative control and TM served as the positive control. A measure of 10 μL of Cms (10^6^ CFU/mL) was added to each well. The plate was incubated 24 h at 28 °C. The lowest concentration of each sample showing a clear aspect served as the MIC [26].

#### 2.7.3. In Vivo Experiments

Fresh potato tubers without wounds were carefully selected for the study. The potato tubers were washed with distilled water and cut lengthwise [27]. The pieces of the potato tubers were soaked in 1% NaClO for 1 min, surface sterilized with 75% ethanol, and rinsed twice with sterile distilled water. DHQ (2 mg) was dissolved in 1% DMSO to gain a 200 μg/mL solution. The positive control was 200 μg/mL TM, and the negative control was 1% DMSO.

##### Protective Assay

A measure of 1 mL of the sample solution was evenly sprayed onto longitudinal sections of the potato tubers. After 2 h, the longitudinal sections were dipped for 15 min in Petri dishes, containing 20 mL Cms suspension (10^6^ CFU/mL). Then, the pieces were placed in plastic containers to incubate at 28 °C. The preventive efficiency was calculated using Formula (1) on the fifth day.

Control efficiency (%) = (C − T)/C × 100%(1)

In the above formula, C is the decayed area, as a percentage of the total longitudinal section area in the negative control group. T is the decayed area as a percentage of the total longitudinal section area in the treatment group.

##### Curative Assay

The longitudinal sections of the potato tubers were dipped in 20 mL Cms suspension (10^6^ CFU/mL) in Petri dishes for 15 min. After 2 h, 1 mL of the sample solution was evenly sprayed onto the longitudinal sections. Then, the pieces were placed in plastic containers to incubate at 28 °C. The curative efficiency was calculated using Formula (1) on the fifth day.

### 2.8. Cell Membrane Damage Assessments

#### 2.8.1. Determination of Membrane Potential

The MP of the Cms was determined according to the method of Wang et al. [28]. Cms cells in the logarithmic phase were centrifuged for 10 min at 4000 rmp, washed twice with PBS and resuspended in PBS to OD_600nm_ = 0.7. The resuspended suspensions were treated with different concentrations of DHQ (1/2MIC, MIC and 2MIC). Treatment with 1% DMSO was used as the control. All the groups were incubated for 12 h at 28 °C. The Cms suspensions (1.5 mL) were incubated with 20 μmol/L rhodamine 123 for 30 min in the dark. The cells were centrifuged, washed twice and resuspended in 1.5 mL PBS. Setting the wavelengths of 480 nm (excitation) and 535 nm (emission), rhodamine fluorescence was measured using a fluorescence spectrophotometer (F7100, Hitachi Instruments (Shanghai) Co., Ltd., Shanghai, China).

#### 2.8.2. Cell Membrane Integrity Analysis

Cell membrane integrity was analyzed following the PI staining assay method [29]. The sample preparation was the same as that described in Section 2.8.1. A measure of 5 μL of PI staining was mixed with the 95 μL Cms suspensions; these were incubated for 30 min away from the light. Then, the cells were washed twice and resuspended in PBS. Setting the wavelengths to 536 nm (excitation) and 539 nm (emission), the fluorescence values were measured in a fluorescence spectrophotometer.

#### 2.8.3. Determine the Leakage of Nucleic Acids and Proteins

The Cms suspension was centrifuged at 4000 rmp (10 min), washed twice with PBS and resuspended in PBS to OD_600nm_ = 0.5. DHQ was added to the resuspended suspension to obtain solutions with 1/2MIC, MIC and 2MIC. Cms cells treated with 1%DMSO were used as the control. All the groups were incubated for 9 h at 28 °C. After centrifugation, supernatants were obtained. The absorbances of the supernatants were measured at 260 nm and 280 nm [30], respectively, using a microplate reader (BioTek Instruments, Inc., Winooski, VT, USA).

### 2.9. Statistical Analysis

Only the data of DIZs of compounds **1**–**5** and in vivo experiments were analyzed using the T-test, and the remaining data were analyzed using a one-way ANOVA test using the SPSS 16.0 software. *p* < 0.05 indicated a significant difference. All the figures were made using the OriginPro 2018.

## 3. Results and Discussion

### 3.1. Antibacterial Potential of Extracts of Different Polarity from FPO

**Figure 1:**
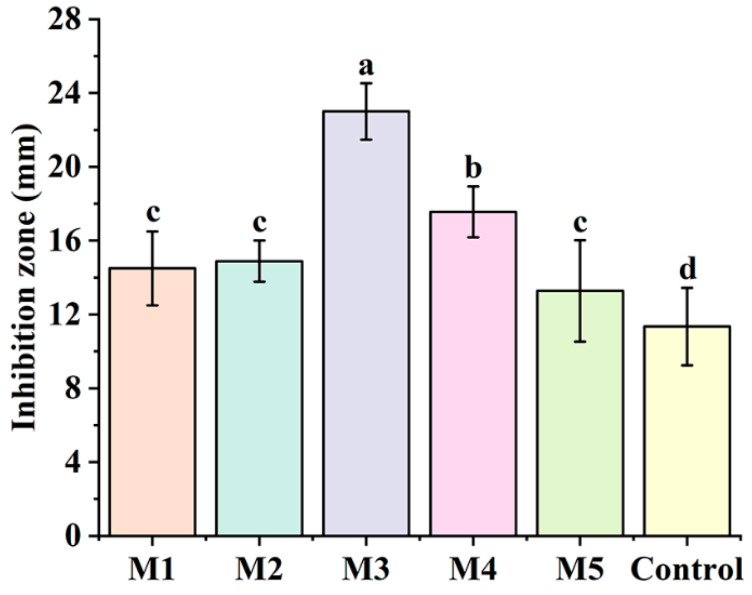
The antibacterial potential of five partition extracts. M1, M2, M3, M4 and M5 were the petroleum ether phase, the chloroform phase, the ethyl acetate phase, the *n*-butanol phase and the water phase, respectively. The concentration of the Cms suspension was 10^6^ CFU/mL. The control was treated with 60% DMSO. The data were analyzed using a Duncan test in one-way ANOVA. Different letters represent significant differences (*p* < 0.05), n = 9.

The DIZ values of five partition extracts (M1–M5) are presented in Figure 1. The five partition extracts were found to have significant (*p* < 0.05) antibacterial activity against Cms. The antibacterial effects of the five partition extracts declined in the following order: M3 (23.00 mm) > M4 (17.56 mm) > M2 (14.88 mm) > M1 (14.50 mm) > M5 (13.28 mm). Compared with the other four phases, M3 showed the highest antibacterial activity. Thus, M3 (EtOAc phase) was selected to further isolate the active compounds.

### 3.2. Active Compounds of EtOAc Phase from FPO

A total of five active compounds were separated using M3. Compounds **1**–**5** of ^1^H NMR, ^13^C NMR and ESI-MS spectra are displayed in Figures S2–S16.

Using the ESI-MS data of **1**, its molecular formula was established as C_16_H_10_O_8_, indicating twelve degrees of unsaturation. The ^13^C NMR spectrum revealed sixteen carbon signals, including two CH_3_, two CH and twelve C. Furthermore, the ^1^H NMR spectrum showed signals classified as two hydroxyl protons at δ_H_ 10.76 (2H, brs, 4-OH and 4′-OH), two aromatic protons at δ_H_ 7.51 (2H, s, H-5 and H-5′) and two oxygenated methines at δ_H_ 4.04 (6H, s, 3-OCH_3_ and 3′-OCH_3_).

The molecular formula of **2** was established as C_22_H_20_O_13_ by ESI-MS at *m*/*z* 515.08039 [M+Na]^+^, which indicated that its degree of unsaturation was thirteen. The ^13^C NMR spectrum showed carbon signals, containing two CH_3_ (two oxygenated), one CH_2_ (one oxygenated), seven CH (five oxygenated) and twelve C (two carbonyl carbons and six oxygenated). In addition, the ^1^H NMR spectrum revealed signals assigned to five hydroxyl protons at δ_H_ 10.87, δ_H_ 5.47, δ_H_ 5.16, δ_H_ 5.15 and δ_H_ 4.58, two aromatic protons at δ_H_ 7.82 and δ_H_ 7.54, two oxygenated methines at δ_H_ 4.09 and δ_H_ 4.05, and protons of sugar moiety at δ_H_ 5.07 and δ_H_ 3.71–3.23.

The ESI-MS analysis recorded a molecular ion peak for compound **3** at *m*/*z* 305.06543 [M+H]^+^, enabling the calculation of its molecular formula as C_15_H_12_O_7_, which possesses ten degrees of unsaturation. The ^13^C NMR analysis of **3** was carried out in Methanol-*d*4, which showed fifteen carbon signals, including seven CH and eight C. The ^1^H NMR spectrum displayed two *ortho*-coupled aromatic protons at δ_H_ 6.85 and δ_H_ 6.81. The protons at δ_H_ 4.91 and δ_H_ 4.51 presented large coupling with *J* = 11.4 Hz.

The molecular ion peak of **4** at *m*/*z* 153.01881 [M–H]^–^ was assigned as molecular formula C_7_H_6_O_4_, which indicated five degrees of unsaturation. The ^13^C NMR spectrum presented three CH and four C signals. Moreover, the carbon signal at δ_C_ 167.34 was attributed to carbonyl carbon. The ^1^H NMR data displayed one carboxyl proton at δ_H_ 12.32, two hydroxyl protons at δ_H_ 9.67 and δ_H_ 9.29, and three aromatic protons at δ_H_ 7.35, δ_H_ 7.29 and δ_H_ 6.79. Moreover, the two aromatic protons at δ_H_ 7.29 and δ_H_ 6.79 were two *ortho*-coupled aromatic protons.

For compound **5**, its molecular formula C_15_H_10_O_7_ was deduced by ESI-MS at *m*/*z* 301.03552 [M–H]^−^, indicating eleven degrees of unsaturation. The ^13^C NMR data of **5** displayed five CH and ten C signals. The ^1^H NMR spectrum of **5** revealed five aromatic proton signals at δ_H_ 7.73, δ_H_ 7.63, δ_H_ 6.89, δ_H_ 6.38 and δ_H_ 6.18. Furthermore, protons at δ_H_ 7.63 and at δ_H_ 6.89 were two *ortho*-coupled aromatic protons with coupling constant of 8.4 Hz.

Comparing the NMR and ESI-MS data with data from the literature, these five known compounds, **1**–**5** were elucidated as 3,3′-di-*O*-methylellagic acid (PubChem CID:5488919) [31], 3,3′-di-*O*-methylellagic acid-4-*O*-*β*-*D*-glucopyranoside (PubChem CID:46918077) [32], dihydroquercetin (PubChem CID:439533) [33], protocatechuic acid (PubChem CID:72) [34] and quercetin (PubChem CID:5280343) [33], respectively.

**Figure 2:**
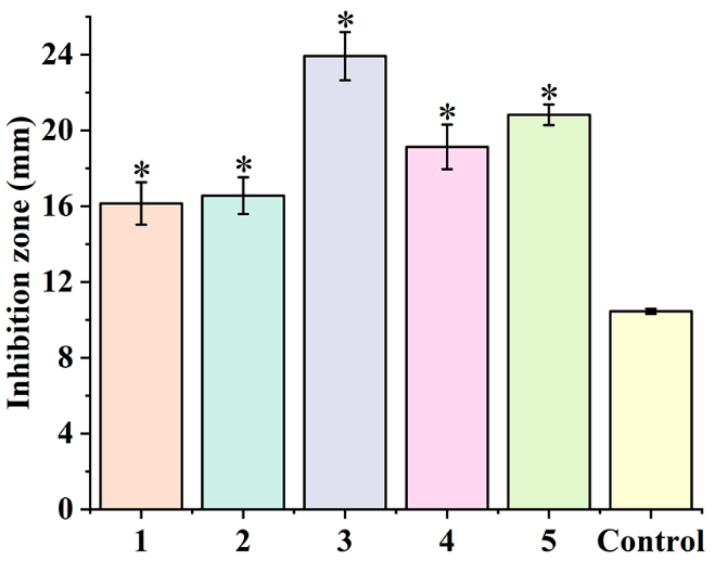
The diameter of the inhibition zone of compounds **1**–**5**. The concentration of compounds **1**–**5** was 8 mg/mL. The control was treated with 60% DMSO. The concentration of Cms suspension was 10^6^ CFU/mL. *t*-test was performed in the experimental group and the control group, respectively. The * indicated a significant difference between the experimental group and the control group (*p* < 0.05), n = 4.

To screen the most active compound, the antibacterial effects of compounds **1**–**5** were determined using the agar well-diffusion method. As shown in Figure 2, compounds **1**–**5** displayed good antibacterial activities against Cms. Especially, compound **3** had the strongest antibacterial activity. Antibacterial activity of above five compounds decreased in the following order: compound **3** (23.92 mm) > compound **5** (20.82 mm) > compound **4** (19.13 mm) > compound **2** (16.56 mm) > compound **1** (16.14 mm). Therefore, compound **3** was selected to investigate the antibacterial activity and cell membrane damage.

### 3.3. Antibacterial Activity of DHQ against Cms In Vitro and In Vivo

#### 3.3.1. DIZ of DHQ

**Figure 3:**
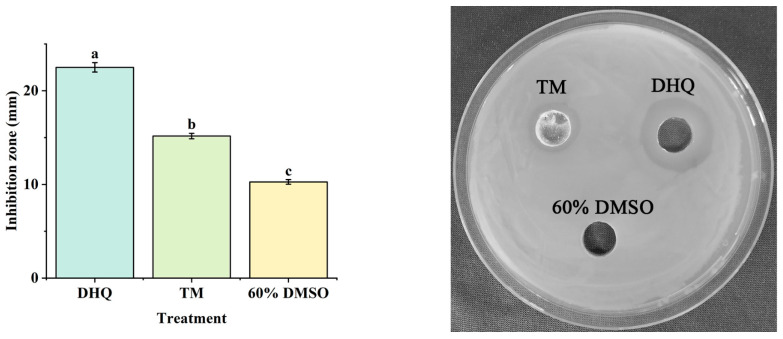
The diameter of inhibition zone of 15 mg/mL DHQ. The negative control and positive control were, respectively, treated with 60% DMSO and 15 mg/mL thiophanate-methyl (TM). The concentration of Cms suspension was 10^8^ CFU/mL. Different letters represent significant differences (*p* < 0.05, one-way ANOVA and Duncan test), n = 3.

TM is a broad-spectrum bactericide that can be used for controlling diseases in crops. TM is generally used to control potato ring rot [35]. Thus, TM was taken as a positive drug in this study. Then, the DIZs of DHQ and TM were compared using the agar well-diffusion method. Compared with the positive control (about 15.17 mm for DIZ), DHQ exhibited a significantly (*p* < 0.05) stronger antibacterial activity against Cms with a DIZ of 22.50 mm (Figure 3).

#### 3.3.2. MIC of DHQ

When the concentrations of DHQ ranged from 10 to 0.313 mg/mL, the samples exhibited a clarity that was equivalent to MIC (0.313 mg/mL) of TM (Table 1). As the concentrations of DHQ decreased, the samples gradually became turbid (Table 1). Specifically, the turbidity at the concentrations of 0.04~0.01 mg/mL was identical to the turbidity of 3% DMSO (Table 1). Therefore, the MIC of DHQ was 0.313 mg/mL. Jeong et al. [36] reported that the MIC of DHQ against vancomycin-resistant *Enterococcus faecalis* was 0.512 mg/mL. Furthermore, Fongang et al. [37] found that the MIC of DHQ against *E. coli*, *Proteus vulgaris*, *Providencia stuartii*, *Staphylococcus aureus* and *Candida albicans* was 0.63 mg/mL. Thus, the DHQ was more sensitive to Cms than the several other bacteria listed here.

#### 3.3.3. In Vivo Antibacterial Effects on Cms

**Figure 4:**
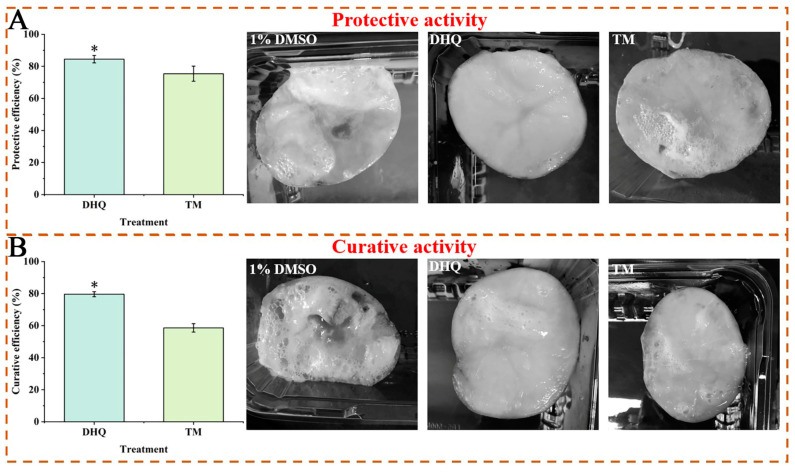
The protective activity (**A**) and curative activity (**B**) of 200 μg/mL DHQ against Cms in vivo. The positive control was 200 μg/mL thiophanate-methyl (TM) and the negative control was 1% DMSO. The data were analyzed using *t*-test. The * represent significant differences (*p* < 0.05), n = 3.

As presented in Figure 4A, DHQ exhibited good protective efficiency (84.49%) against Cms in vivo at 200 μg/mL, which exceeded that of TM (75.39%). Simultaneously, at the same concentration, DHQ also displayed good curative efficiency (79.63%) in controlling Cms; this was superior to that of TM (58.61%), as shown in Figure 4B.

TM potentially possesses genotoxicity and its metabolite (carbendazim) has been considered to be a possible human carcinogen [38,39]. In addition, TM can lead to pollution and enrichment in related crops [40]. The toxicity and genotoxicity of DHQ have been studied. Booth and DeEds [41] have reported that the long-term feeding of DHQ to albino rats at a dietary level of 1% is nontoxic. Zhanataev et al. [42] have reported that DHQ did not cause DNA damage to the bone marrow, blood, liver or rectal cells of mouse in DNA-comet assays. Meanwhile, DHQ was not found to affect the chromosomal aberrations of mouse bone marrow cells at single administration of 1.5 mg/kg, 150 mg/kg and five times administration of 1.5 mg/kg [42]. In addition, DHQ can be commonly found in various plants, including onions and milk thistle, etc. [43]. In Russia, DHQ has regulatory approval of using as a food additive and dietary supplement ingredient [44]. There are concerns about long-term effects of chemical bactericides on humans and the environment. Therefore, in order to obtain a series of safe and efficient compounds against Cms, DHQ could be served as a potential lead molecular in the future. In order to offer a theoretical basis for application, the antibacterial mechanism of DHQ was subsequently studied.

### 3.4. Effects of DHQ on Cell Membrane

DHQ has the basic skeleton of C6-C3-C6, belonging to the family of flavonoids, which are the most prevalent of polyphenols [45]. The antibacterial activity of polyphenols is associated with hydroxy group on aromatic rings and on oxygen-substituted ring [45]. Owing to the interaction of hydroxy radicals of the polyphenols with the cell membrane, polyphenols can accumulate on the surface of bacteria, causing a series of changes in the cell membrane [46]. Therefore, the MP, cell membrane integrity and the leakage of nucleic acids and proteins were determined to enable the assessment of cell membrane damage.

#### 3.4.1. Effect of DHQ on Membrane Potential

**Figure 5:**
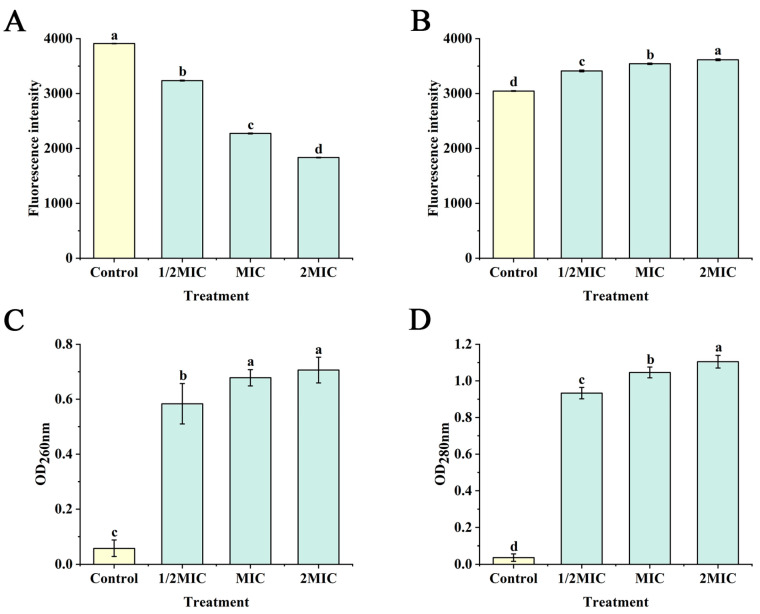
Effects of DHQ on membrane potential (**A**), cell membrane integrity (**B**), leakage of nucleic acids (**C**) and proteins (**D**) of Cms. The control was treated with 1% DMSO. Different letters represent significant differences (*p* < 0.05, one-way ANOVA and Duncan test), n = 3.

The changes in MP of Cms, treated using DHQ, are presented at Figure 5A. Compared with the control, the fluorescence intensity of rhodamine 123 markedly (*p* < 0.05) decreased in the groups of 1/2MIC, MIC and 2MIC, indicating a reduction in the MP (Figure 5A). MP is defined as the potential difference between the two sides of a cell membrane, which is affected by the changes in a cell membrane structure [47]. The reduction in MP implies the occurrence of depolarization of the cell membrane, an important type of membrane damage [47,48]. Thus, this result revealed that DHQ damaged the cell membranes of Cms, causing an obvious depolarization phenomenon in the cell membrane.

#### 3.4.2. Effect of DHQ on Cell Membrane Integrity

PI, a membrane-impermeable dye, was used to evaluate the integrity of the cell membrane; PI can enter into compromised cell membranes to interact with their DNA. In this study, the fluorescence intensity values for Cms treated with DHQ were significantly (*p* < 0.05) higher than the control (Figure 5B). When PI is excluded from cells and cannot interact with DNA, the fluorescence intensity is weak [49]. Therefore, those differences suggested that DHQ had the ability to damage the cell membrane integrity of Cms, leading to the reduction in Cms survival.

#### 3.4.3. Effect of DHQ on Leakage of Nucleic Acids and Proteins

The leakage of nucleic acids and proteins can reflect disruptions in the cell membrane structure [48]. In Figure 5C,D, compared with the control, the values of OD_260nm_ and OD_280nm_ significantly (*p* < 0.05) increased in the groups of 1/2MIC, MIC and 2MIC; this implied that considerable amounts of nucleic acids and proteins leaked from the Cms cells. These results further revealed that the cell membrane structures of Cms were disrupted by DHQ. Nucleic acids and proteins play a critical part in maintaining the normal life activities of bacteria, such as the transmission of genetic information and provision of structural functions [50]. Therefore, the normal function of Cms may be seriously affected.

## 4. Conclusions

Through sequential extraction and bioassay-guided method, five active compounds were isolated and identified: 3,3′-di-*O*-methylellagic acid (**1**), 3,3′-di-*O*-methylellagic acid-4-*O*-*β*-*D*-glucopyranoside (**2**), dihydroquercetin (**3**), protocatechuic acid (**4**) and quercetin (**5**). Of the five compounds mentioned, compound 3 (DHQ) had the strongest antibacterial activity against Cms in comparison with the other compounds. Moreover, DHQ exhibited excellent antibacterial activity against Cms in vitro and in vivo. The DIZ, MIC, protective efficiency and curative efficiency were 22.50 mm, 0.313 mg/mL, 84.49% and 79.63%, respectively, exceeding those of TM. The results of the cell membrane damage assessments suggested that DHQ had the ability to disrupt the cell membranes by inducing the depolarization of the cell membranes, damaging their integrity and further promoting the leakage of nucleic acids and proteins at a cellular level. This study only assessed cell membrane damage of DHQ at the cellular level, not at the molecular level by using the model membrane. Subsequently, future research must shift its focus towards comprehending the membrane-acting mechanism of DHQ using the model membrane from a molecular perspective. Using DHQ as a principal molecule, this further research will aim to design a novel series of antibacterial compounds which act against Cms.

## Figures and Tables

**Table 1:** The MIC of DHQ against Cms.

Strain	Concentrations of DHQ (mg/mL)											3% DMSO	MIC of TM
	10	5	2.5	1.25	0.625	0.313	0.156	0.08	0.04	0.02	0.01		
Cms	−	−	−	−	−	−	+	+	+ + +	+ + +	+ + +	+ + +	0.313 mg/mL

Thiophanate-methyl (TM) and 3% DMSO were, respectively, served as positive control and negative control. “−” represents the sample showing clear; “+” represents the sample showing turbid; “+ + +” represents the sample showing entirely turbid.

## Data Availability

The data used to support the findings of this study can be made available by the corresponding author upon request.

## Supplementary Materials

The following supporting information can be downloaded at: https://www.mdpi.com/article/10.3390/foods13010023/s1, Figure S1: The isolation scheme of active compounds from ethyl acetate phase. Figures S2–S16 show the ^1^H NMR spectra, ^13^C NMR spectra and primary mass spectra of compounds **1**–**5**, respectively.
