# Supplementary material for: Antibacterial Activity of Dihydroquercetin Separated from Fructus Polygoni orientalis against Clavibacter michiganensis subsp. sepedonicus via Damaging Cell Membrane
Source: Foods. 2023 Dec 20;13(1):23. doi: 10.3390/foods13010023 (PMC10778462; doi:10.3390/foods13010023)
Supplement: Supplementary file 1 [file foods-13-00023-s001.zip › foods-2758826-supplementary.pdf]

Supplementary Information

Antibacterial Activity of Dihydroquercetin Separated from Fructus *Polygoni orientalis* against *Clavibacter michiganensis* subsp. *sepedonicus* via Damaging Cell Membrane

Jin Cai [1,*], Shiqin Wang [2,3] and Qi Wang [4]

1 Institute of Applied Chemistry, Shanxi University, Taiyuan 030006, China
2 Morden Research Center for Traditional Chinese Medicine, Shanxi University, Taiyuan 030006, China; wangshiqin12247@163.com
3 The Key Laboratory of Chemical Biology and Molecular Engineering of Ministry of Education,
Shanxi University, Taiyuan 030006, China
4 School of Life Science, Shanxi University, Taiyuan 030006, China; wangqi@sxu.edu.cn
* Correspondence: caijin@sxu.edu.cn

1. The isolation scheme of active compounds from ethyl acetate phase

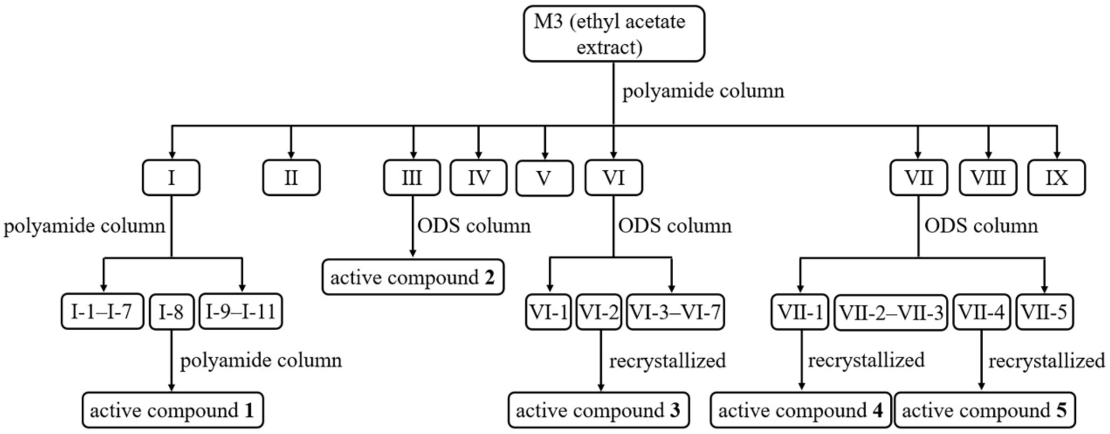

Figure S1. The isolation scheme of active compounds from ethyl acetate phase.

2. Characterizations of compounds **1–5**

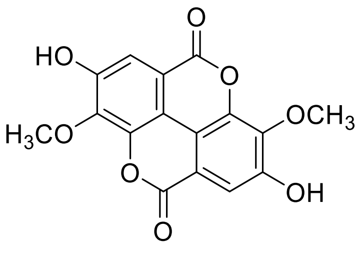

3,3’-di-*O*-methylellagic acid (1):

ESI-MS: *m/z* 331.04480 $[M+H]^+$, *m/z* 329.03036 $[M–H]^-$. ^{1}H NMR (600 MHz, DMSO-d_6): δ 10.76 (2H, brs, 4-OH and 4’-OH), 7.51 (2H, s, H-5 and H-5’), 4.04 (6H, s, 3-OCH_3 and 3’-OCH_3); ^{13}C NMR (151 MHz, DMSO-d_6): δ 158.50 (C-7 and C-7’), 152.25 (C-4 and C-4’), 141.23 (C-2 and C-2’), 140.24 (C-3 and C-3’), 112.13 (C-6 and C-6’), 111.65 (C-1 and C-1’), 111.47 (C-5 and C-5’), 60.97 (3-OCH_3 and 3’-OCH_3).

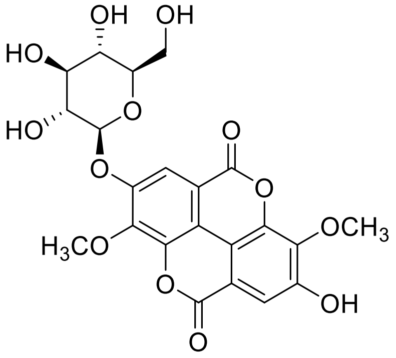

3,3’-di-*O*-methylellagic acid-4-*O*-*β*-*D*-glucopyranoside (2):

ESI-MS: *m/z* 515.08039 $[M+Na]^+$, *m/z* 491.08342 $[M–H]^-$. ^{1}H NMR (600 MHz, DMSO-d_6): δ 10.87 (1H, brs, 4-OH), 7.82 (1H, s, H-5’), 7.54 (1H, s, H-5), 4.09 (3H, s, 3’-OCH_3), 4.05 (3H, s, 3-OCH_3) for aglycone; 5.47 (1H, s, 2”-OH), 5.07 (1H, d, J = 6.0 Hz, H-1”), 5.16 (1H, s, 4”-OH), 5.15 (1H, s, 3”-OH), 4.58 (1H, s, 6”-OH), 3.71–3.23 (6H, m, H-2”, H-3”, H-4”, H-5”, H-6”a and H-6”b) for sugar moiety. ^{13}C NMR (151 MHz, DMSO-d_6): δ 158.32 (C-7’), 158.25 (C-7), 152.73 (C-4’), 151.45 (C-4), 141.64 (C-2’), 141.58 (C-2), 140.87 (C-3’), 140.07 (C-3), 114.04 (C-6’), 112.74 (C-1’), 111.85 (C-6), 111.73 (C-5), 111.50 (C-5’), 111.07 (C-1), 101.18(C-1”), 77.15 (C-5”), 76.37 (C-3”), 73.24 (C-2”), 69.36 (C-4”), 61.55 (3’-OCH_3), 60.91 (3-OCH_3), 60.41 (C-6”).

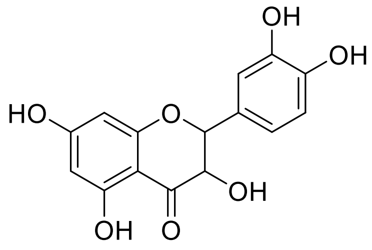

dihydroquercetin (3):

ESI-MS: *m/z* 305.06543 $[M+H]^+$, *m/z* 303.05109 $[M–H]^–$. ^{1}H NMR (600 MHz, Methanol-*d*4): δ 6.97 (1H, s, H-2'), 6.85 (1H, d, *J* = 7.8 Hz, H-6'), 6.81 (1H, d, *J* = 7.8 Hz, H-5'), 5.92 (1H, s, H-8), 5.88 (1H, s, H-6), 4.91 (1H, d, *J* = 11.4 Hz, H-2), 4.51 (1H, d, *J* = 11.4 Hz, H-3); ^{13}C NMR (151 MHz, Methanol-*d*4): δ 198.40 (C-4), 168.74 (C-7), 165.30 (C-5), 164.50 (C-9), 147.13 (C-4'), 146.30 (C-3'), 129.85 (C-1'), 120.89 (C-6'), 116.06 (C-5'), 115.86 (C-2'), 101.81 (C-10), 97.30 (C-6), 96.27 (C-8), 85.10 (C-2), 73.65 (C-3).

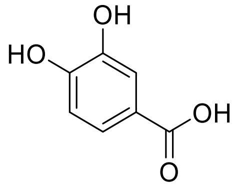

protocatechuic acid (4):

ESI-MS: *m/z* 154.99059 $[M+H]^+$, *m/z* 153.01881 $[M–H]^–$. ^{1}H NMR (600 MHz, DMSO-d_6): δ 12.32 (1H, s, H-COOH), 9.67 (1H, s, 3-OH), 9.29 (1H, s, 4-OH), 7.35 (1H, s, H-2), 7.29 (1H, d, *J* = 8.4 Hz, H-6), 6.79 (1H, d, *J* = 7.8 Hz, H-5); ^{13}C NMR (151 MHz, DMSO-d_6): δ 167.34 (C-1'), 150.03 (C-4), 144.90 (C-3), 121.92 (C-6), 121.67 (C-1), 116.57 (C-2), 115.18 (C-5).

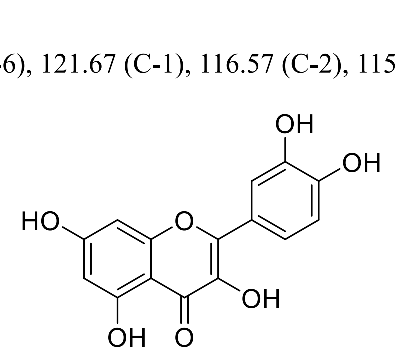

quercetin (5):

ESI-MS: *m/z* 303.05045 $[M+H]^+$, *m/z* 301.03552 $[M–H]^–$. ^{1}H NMR (600 MHz, Methanol-d_4): δ 7.73

(1H, s, H-2'), 7.63 (1H, d, J = 8.4 Hz, H-6'), 6.89 (1H, d, J = 8.4 Hz, H-5'), 6.38 (1H, s, H-8), 6.18 (1H, s, H-6); ^{13}C NMR (151 MHz, Methanol-d4): δ 177.32 (C-4), 165.55 (C-7), 162.49 (C-5), 158.21 (C-9), 148.75 (C-4'), 147.97 (C-3'), 146.21 (C-2), 137.23 (C-3), 124.14 (C-1'), 121.66 (C-6'), 116.21 (C-5'), 115.98 (C-2'), 104.50 (C-10), 99.22 (C-6), 94.39 (C-8).

3. ^{1}H NMR, ^{13}C NMR and ESI-MS data

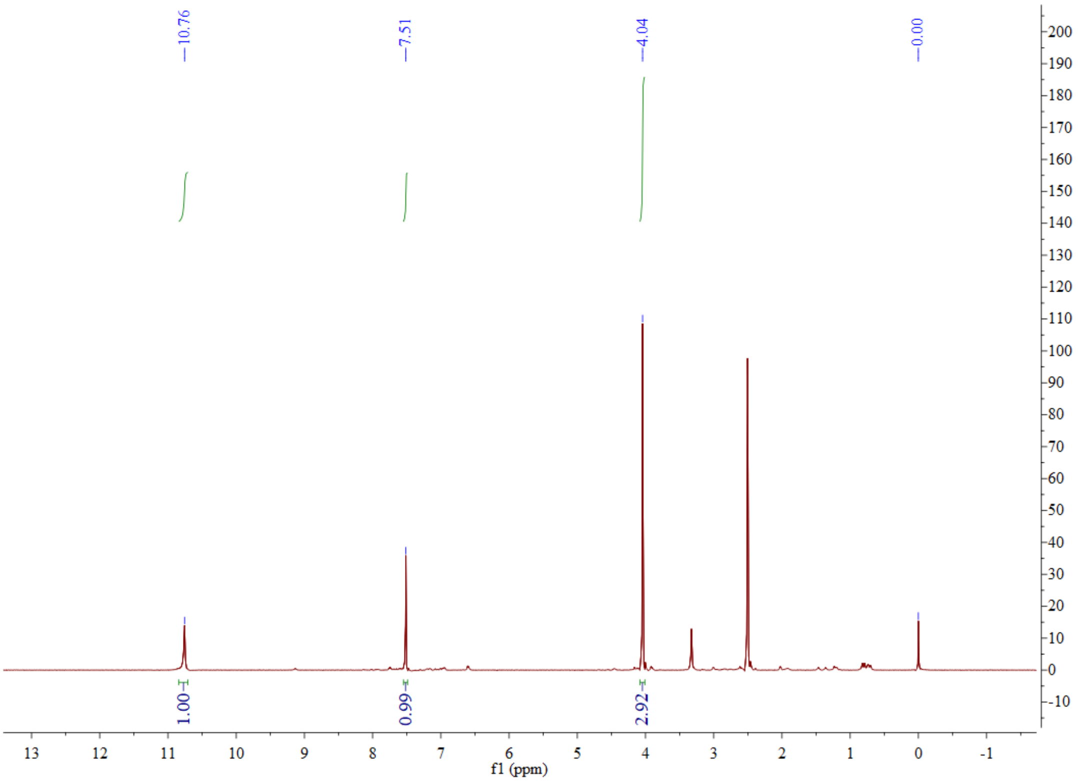

Figure S2. The ^{1}H NMR spectrum of 3,3'-di-*O*-methylellagic acid (**1**).

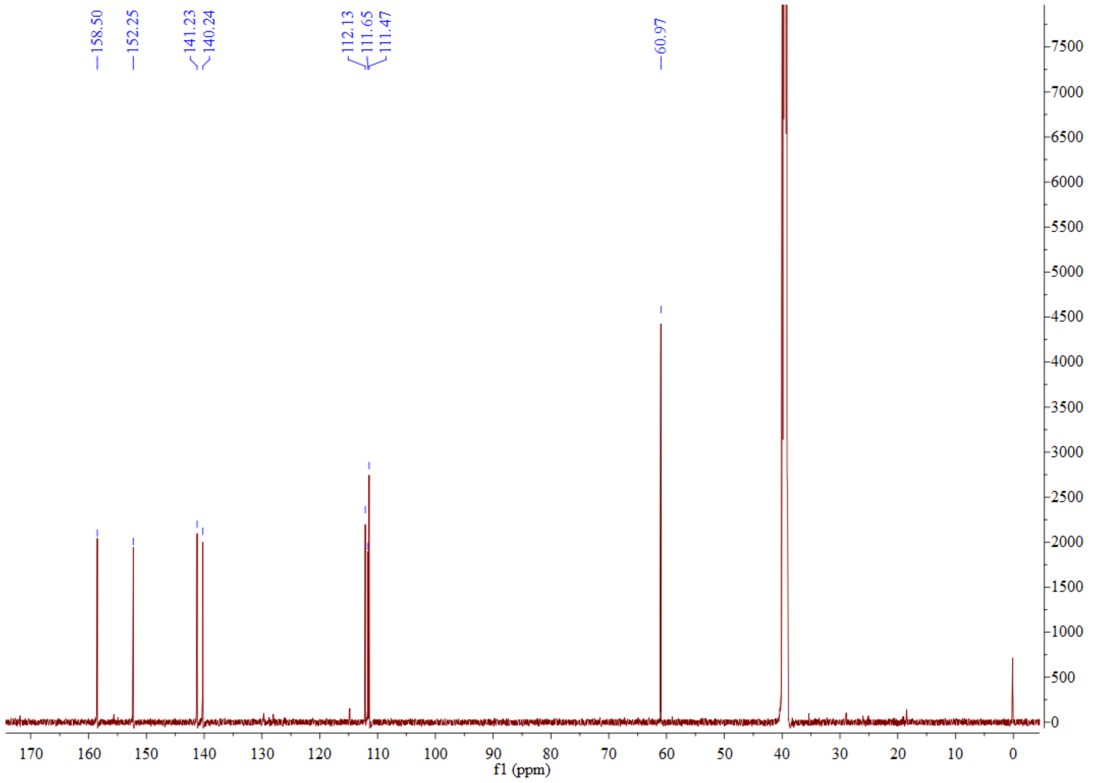

Figure S3. The ^{13}C NMR spectrum of 3,3'-di-*O*-methylellagic acid (**1**).

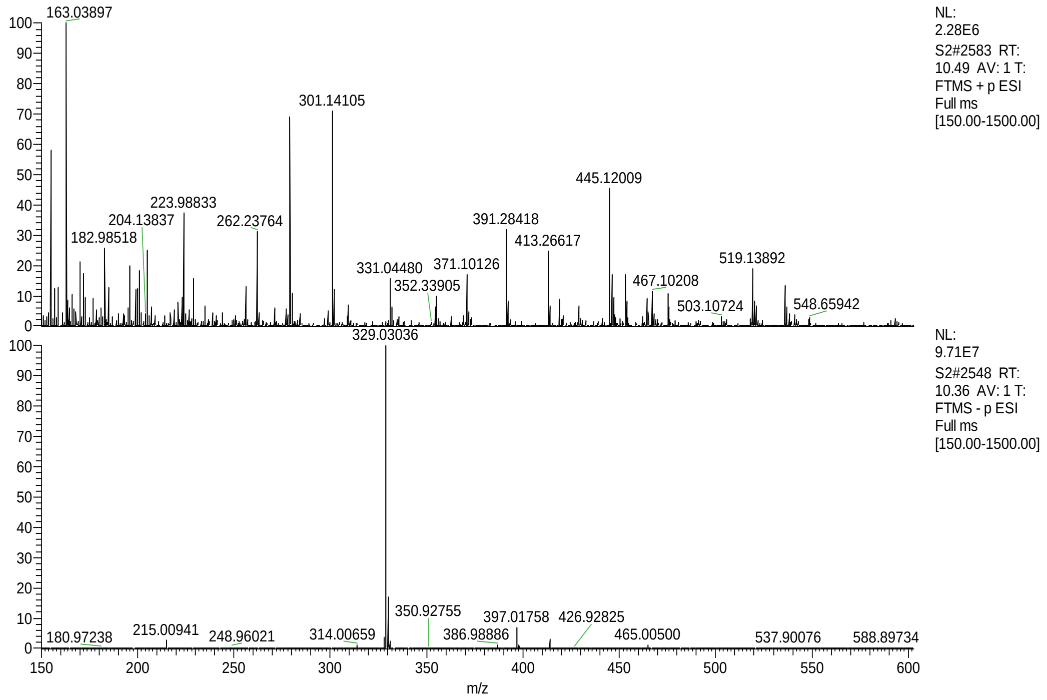

Figure S4. The primary mass spectrum of 3,3'-di-*O*-methylellagic acid (**1**).

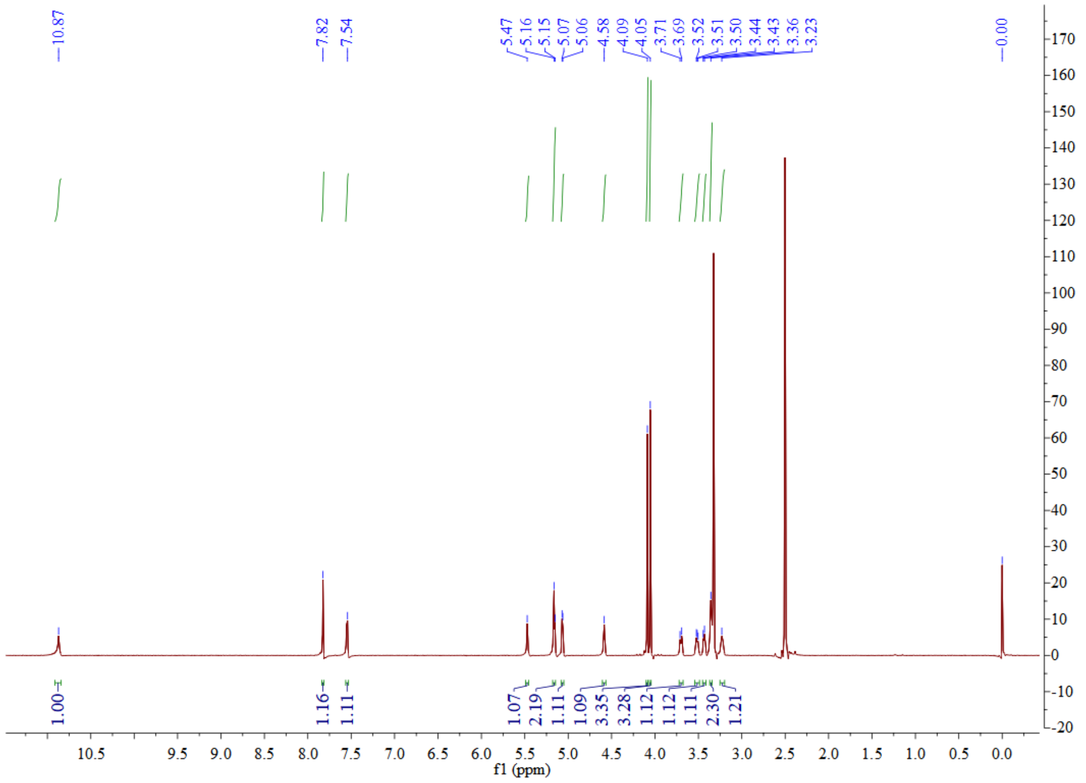

Figure S5. The ^{1}H NMR spectrum of 3,3'-di-*O*-methylellagic acid-4-*O*-*β*-*D*-glucopyranoside (**2**).

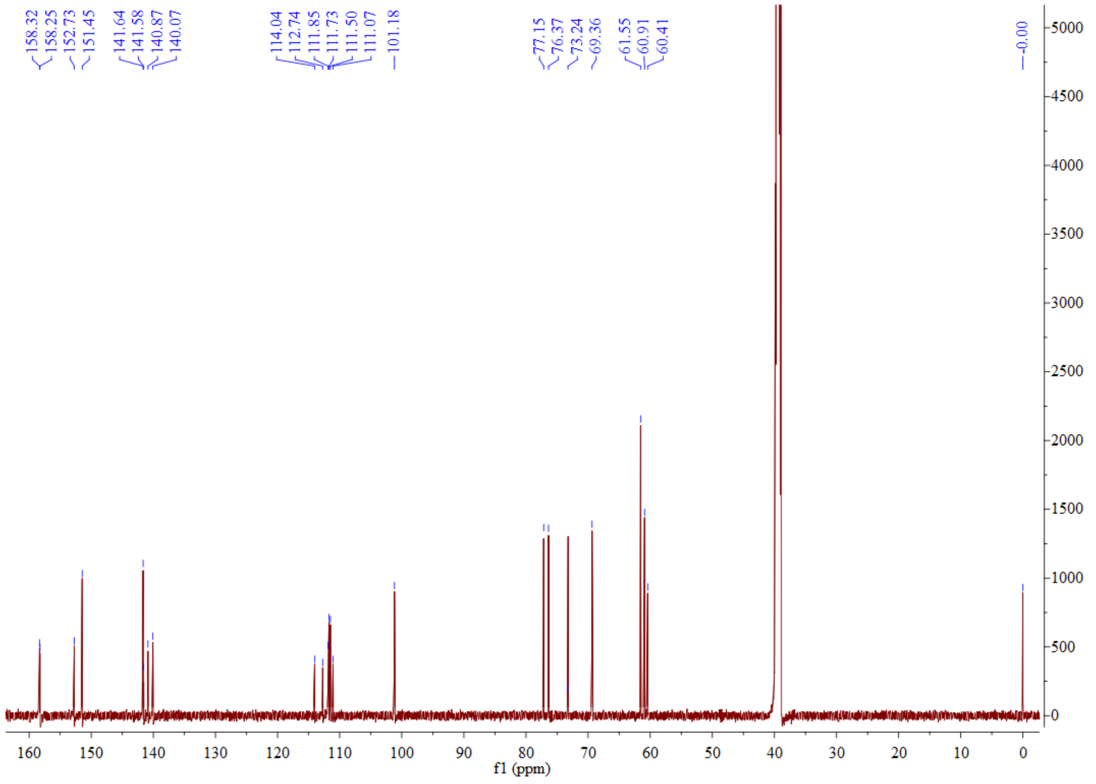

Figure S6. The ^{13}C NMR spectrum of 3,3'-di-*O*-methylellagic acid-4-*O*-*β*-*D*-glucopyranoside (**2**).

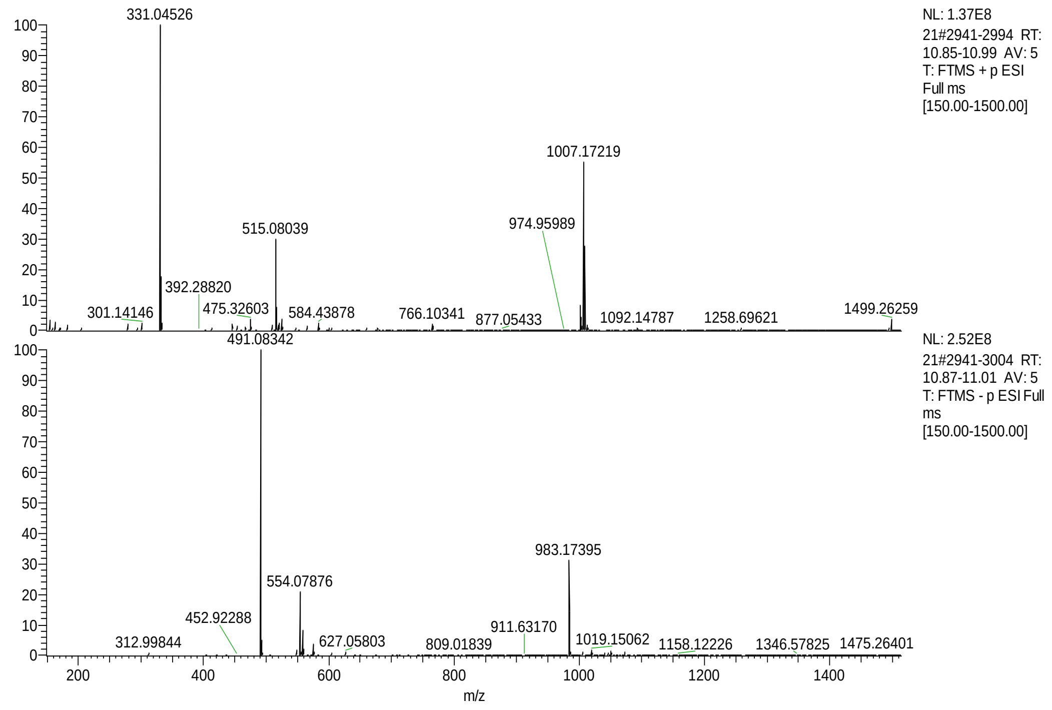

Figure S7. The primary mass spectrum of 3,3'-di-*O*-methylellagic acid-4-*O*-*β*-*D*-glucopyranoside (**2**).

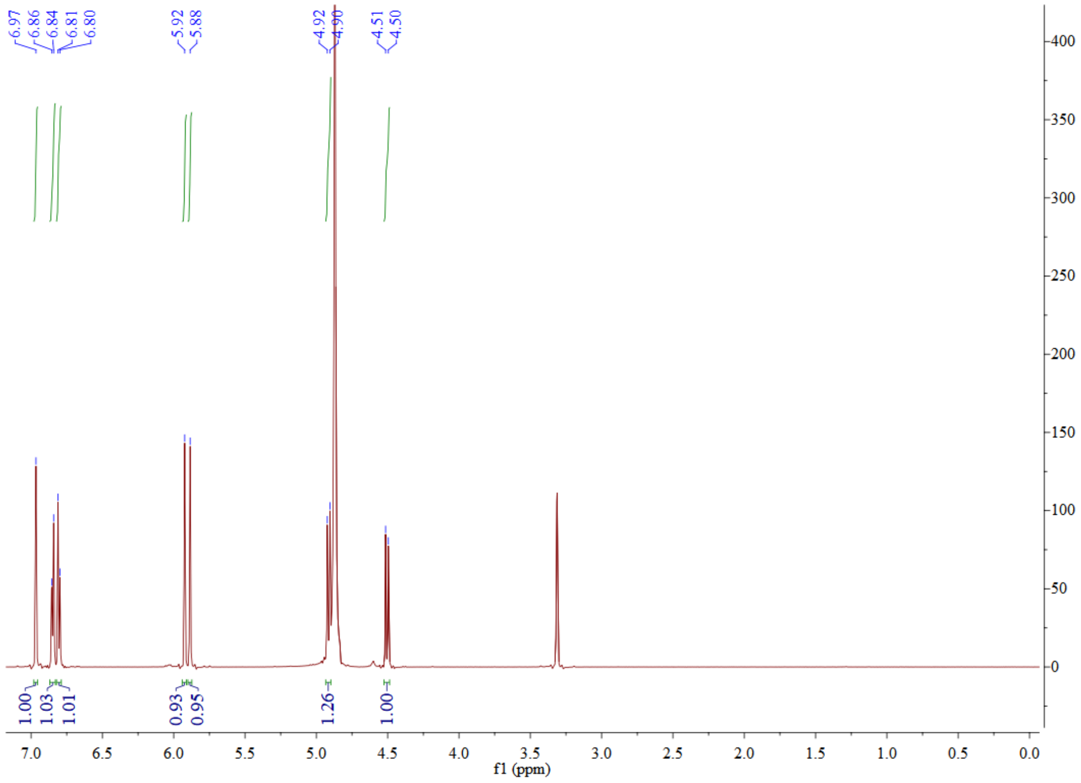

Figure S8. The ^{1}H NMR spectrum of dihydroquercetin (**3**).

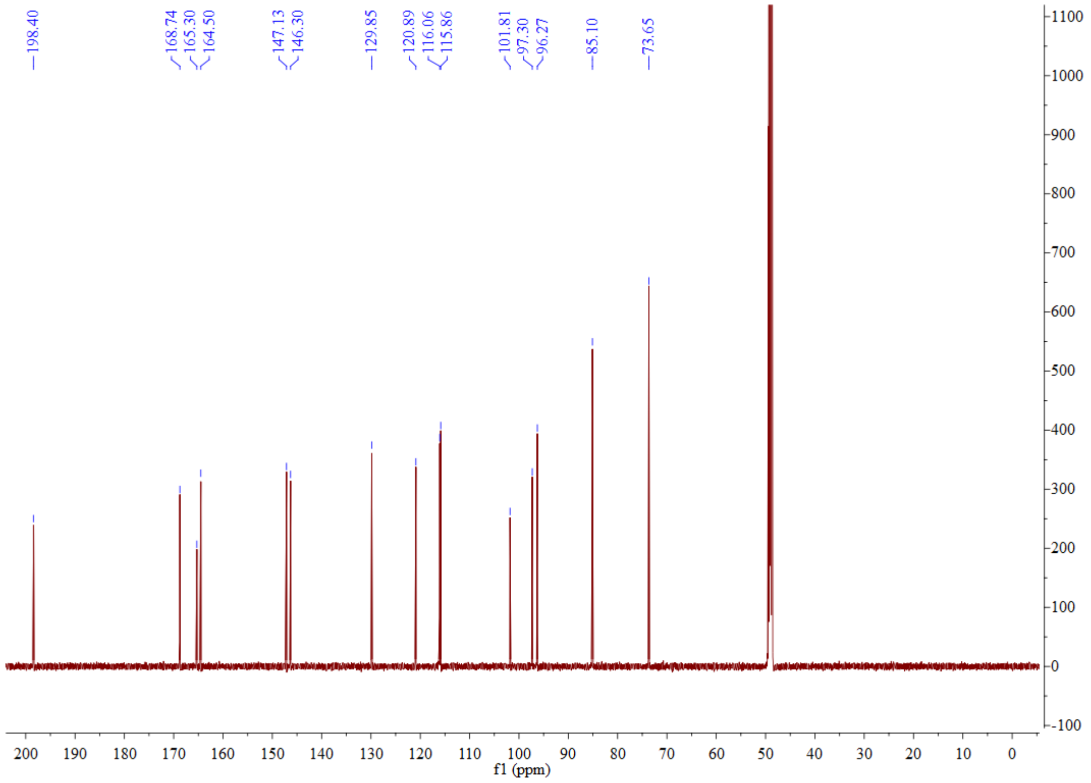

Figure S9. The ^{13}C NMR spectrum of dihydroquercetin (**3**).

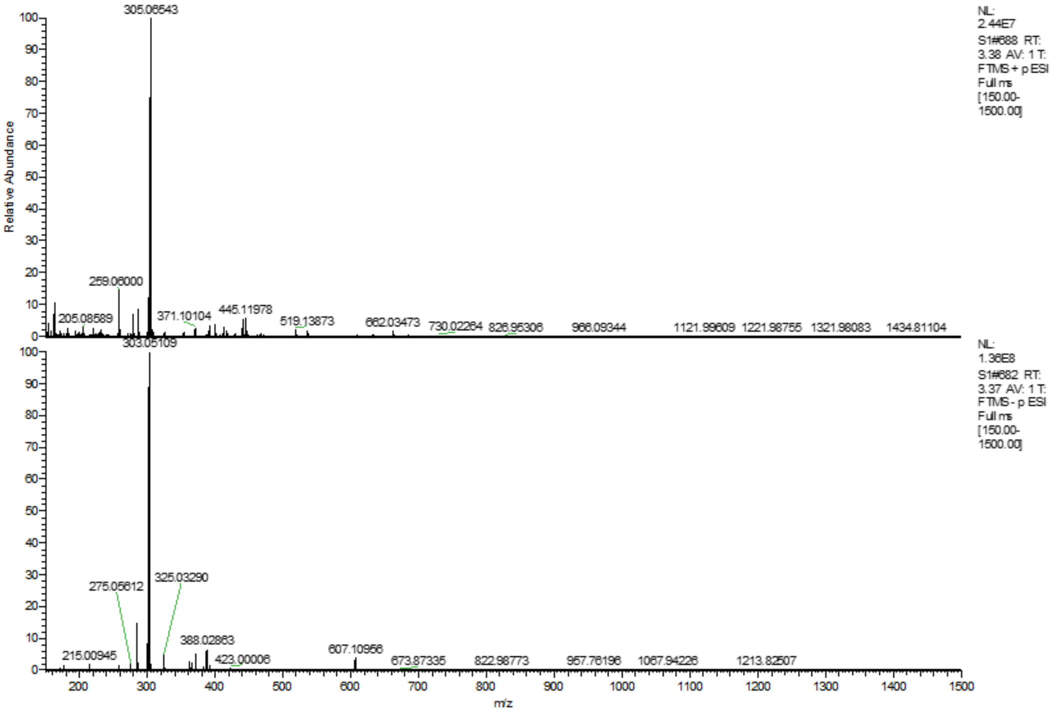

Figure S10. The primary mass spectrum of dihydroquercetin (**3**).

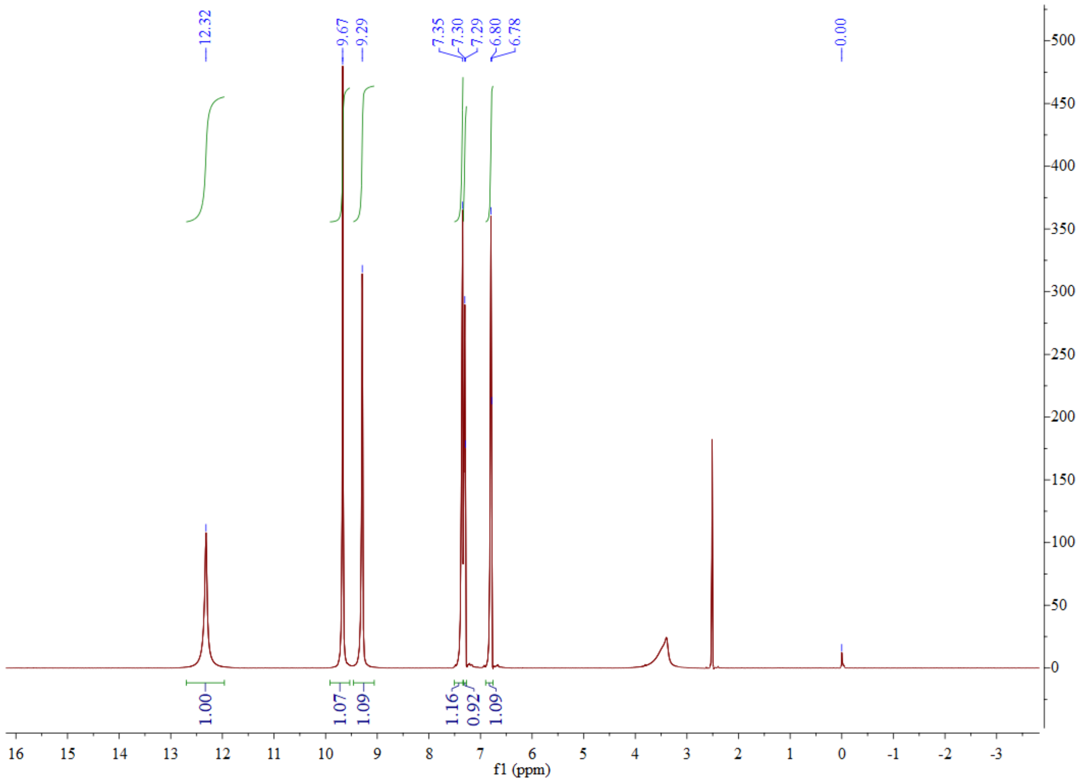

Figure S11. The ^{1}H NMR spectrum of protocatechuic acid (**4**).

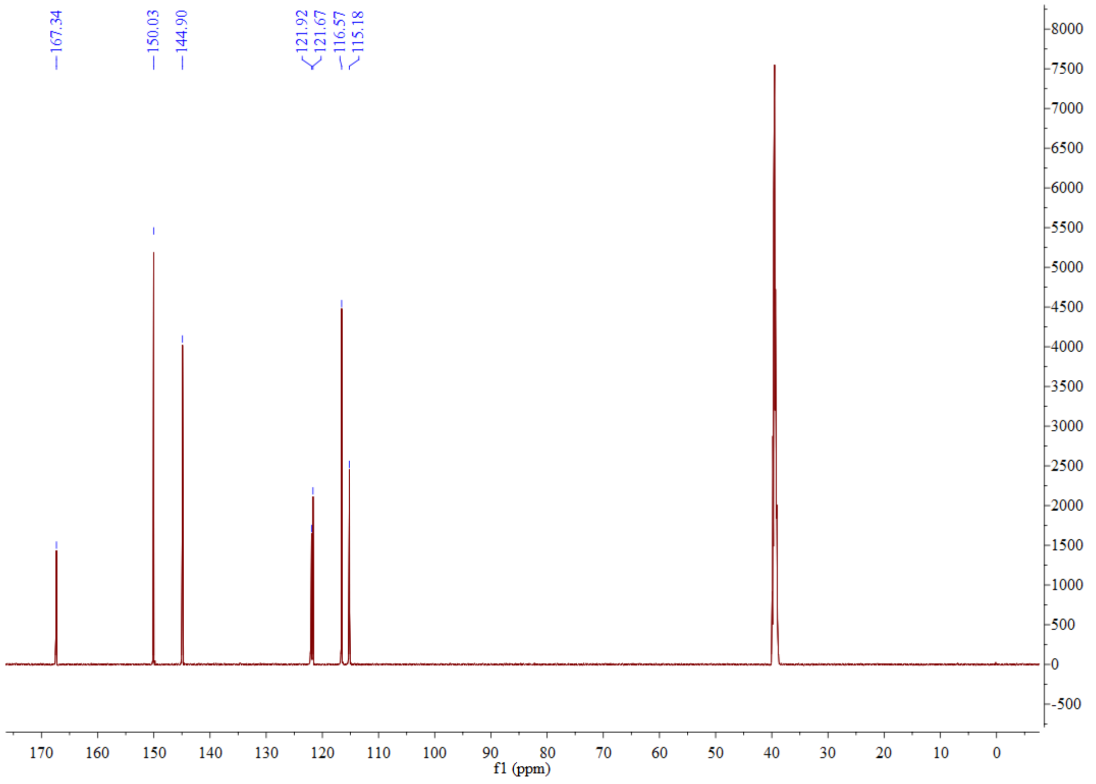

Figure S12. The ^{13}C NMR spectrum of protocatechuic acid (**4**).

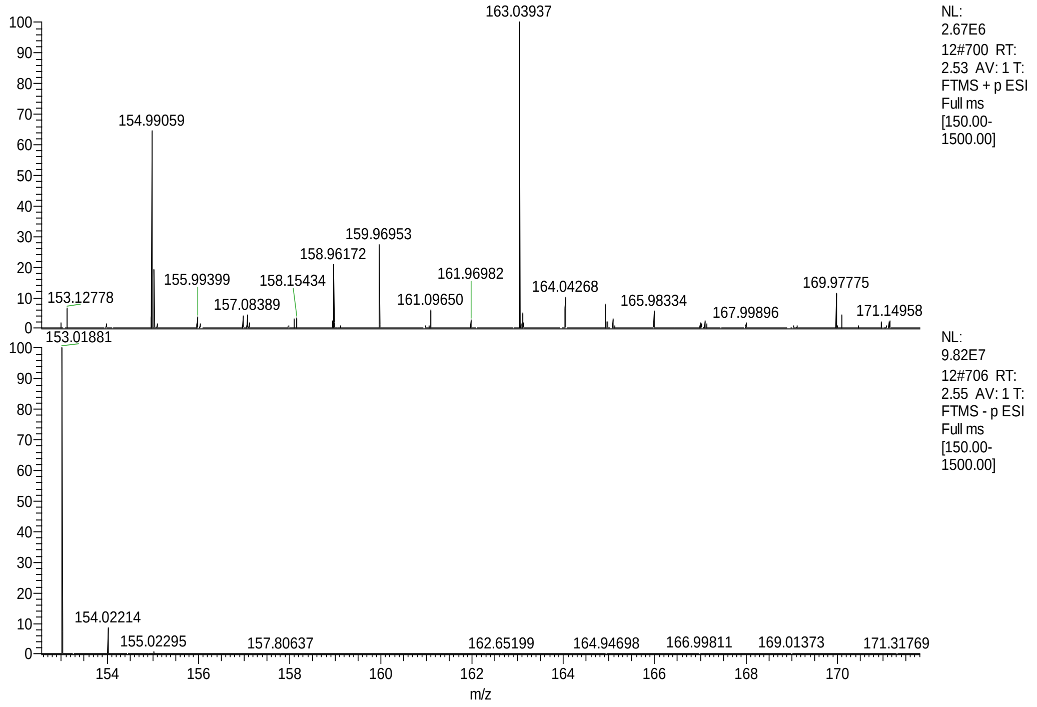

Figure S13. The primary mass spectrum of protocatechuic acid (**4**).

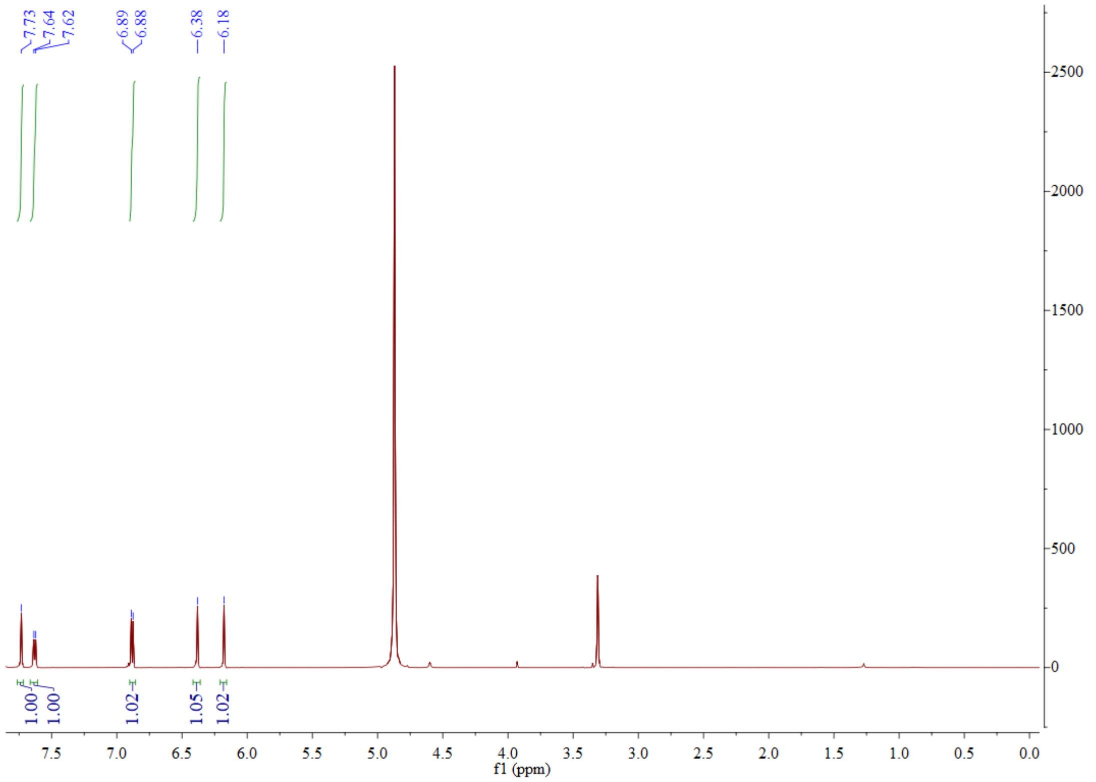

Figure S14. The ^{1}H NMR spectrum of quercetin (**5**).

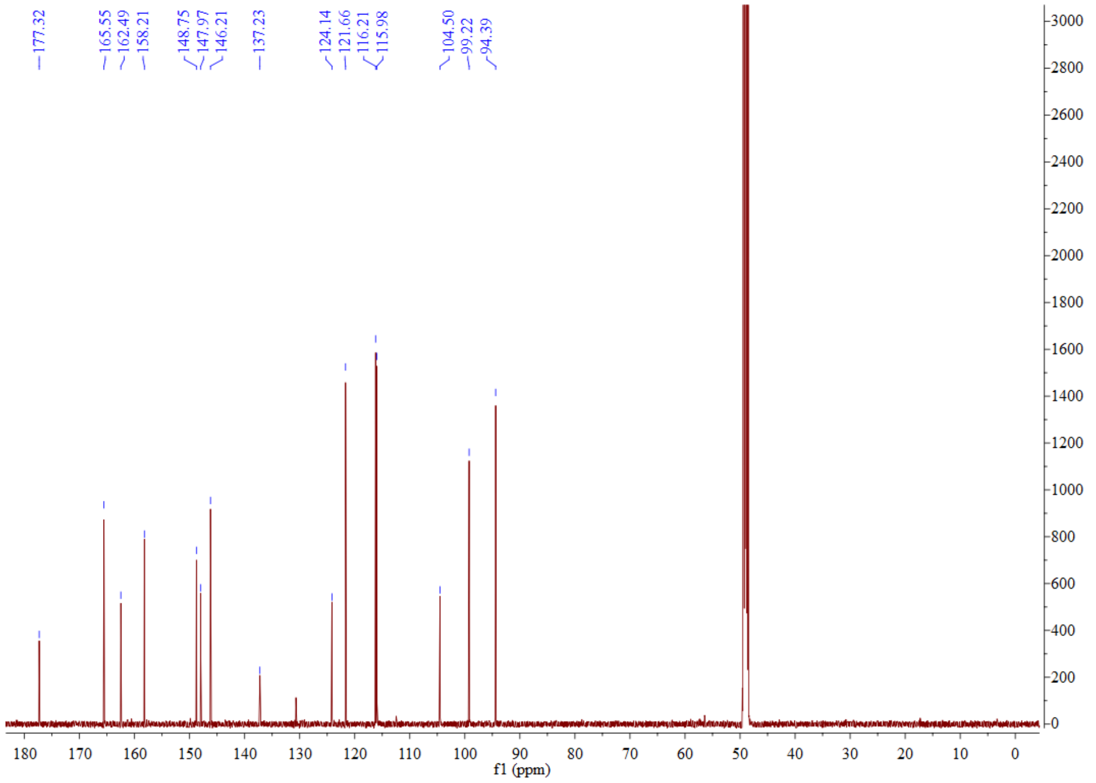

Figure S15. The ^{13}C NMR spectrum of quercetin (**5**).

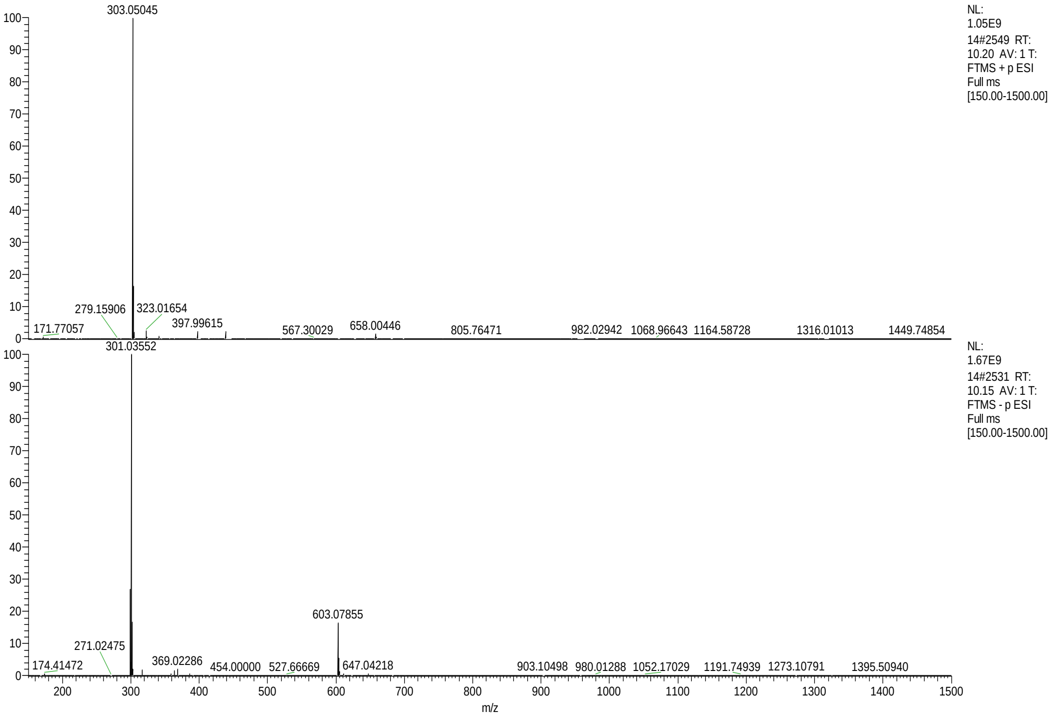

Figure S16. The primary mass spectrum of quercetin (**5**).
